# Neurooncological Rehabilitation in Diffuse Gliomas

**DOI:** 10.7759/cureus.57534

**Published:** 2024-04-03

**Authors:** Levent Tanrikulu, Ulf Seifart

**Affiliations:** 1 Oncology, Klinik Sonnenblick, University of Marburg, Marburg, DEU

**Keywords:** outcome, rehabilitation, physical therapy, glioma, neurooncology

## Abstract

Background: With the further advancement of surgical technology and modern tumor-targeted treatment strategies, longer survival rates can be achieved in diffuse gliomas. Pre- and post-therapeutic physical and cognitive deficits are frequently associated with gliomas. The clinical impact of physical therapy and rehabilitation on neurooncological disorders has not been analyzed consecutively. This study investigates the clinical effect of rehabilitation in patients with diffuse gliomas.

Methods: Patients with surgically and radio-/oncologically treated diffuse gliomas were recruited into this study. They were admitted to an inpatient program for three weeks. The patients underwent physical and occupational therapy, exercise programs, and psychooncological support. The outcome measures included motor strength, mobility, neuropsychological deficits, and tumor localization-dependent symptoms for the statistical determination and comparison of the respective Eastern Cooperative Oncology Group (ECOG) scores on admission and discharge by a two-tailed t-test.

Results: A total of 25 patients (f/m: 11/14) with diffuse gliomas were recruited into the program. Four patients (16%) had CNS WHO grade 2, seven patients (28%) had grade 3, and 14 patients (56%) had grade 4 tumors. Major improvement in motor, cognitive, and functions of the daily activities was achieved in the examined population. Major improvement in motor, cognitive, and neurological functions of the daily activities screened in the admission of all patients was achieved. The comparison of the ECOG scores determined on admission and on discharge showed a statistical significance derived from the undertaken t-test with a p-value <0.05.

Conclusion: We herein show that a clearly defined inpatient rehabilitation protocol significantly enables the improvement of the quality of life of patients with diffuse gliomas. The effectiveness of the exercise program and psychooncological assistance was confirmed by the course of patient-reported functions. Based on the limited number of our patient collective, multicenter studies with broader patient sizes should be performed to confirm our significant results.

## Introduction

Gliomas represent the most common neoplastic disorder of the brain [1]. These tumors originate from the glial cells [1]. The classification of gliomas was revised in the year 2021 [1]. The first-line treatment, depending on the localization of the tumor, is radically but functionally gross total microsurgical resection [2]. In highly eloquent gliomas, the decision for resection or histological confirmation by biopsy has to come individually [2]. The standard of treatment includes postoperative adjuvant radiotherapy, chemotherapy, tumor-treating fields, and in some instances immune and experimental therapies according to study protocols [3,4].

Rehabilitation in patients with diffuse gliomas was rarely analyzed in the literature [5-7]. Common disorders that require neurooncological rehabilitation include patients with resected astrocytomas especially in cerebral areas with eloquent functionality, because these patients have a higher risk of experiencing neurological deficits such as pareses and neuropsychological deficits.

Utilization of the modern treatment paradigm led to the survival of 16.3 months across studies and had a mean cost of 62,602 USD [8]. In other common cancerous diseases such as leukemia and breast, gastrointestinal, and pulmonary cancers, plenty of published data exist, while the literature is lacking in gliomas dealing with the impact of rehabilitation on the quality of life [9]. We herein analyze the clinical effect of inpatient rehabilitation and psychooncological assistance of patients with diffuse gliomas.

## Materials and methods

**Figure 1:**
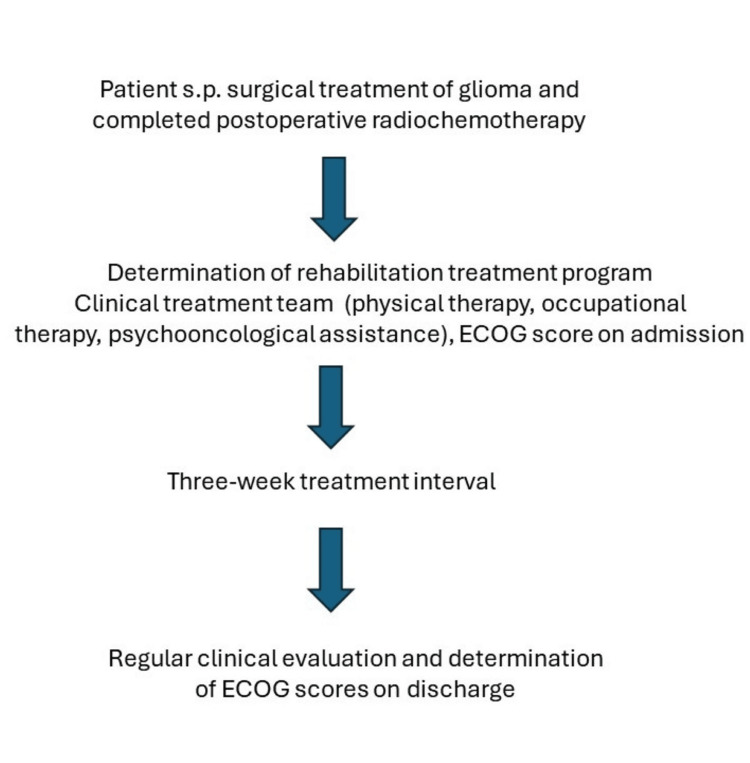
Patient selection flowchart s.p.: status post; ECOG: Eastern Cooperative Oncology Group

In this study, we analyzed 25 patients with diffuse gliomas, who underwent surgery and postoperative radio-/chemotherapy (RCT) between 2021 and 2023 (Table 1). The Medical Ethics Committee at the University of Marburg approved the study protocol (24-77 RS). All of these patients were admitted to our program in the postoperative interval within 12 months after the completion of RCT. The included patients corresponded to a Barthel index ≥60 points in the daily activities [10]. The patients were scheduled after registration through the admission process of the German age insurance (Figure 1). A detailed medical history was obtained, and a neurological examination was performed. After history and examination, the treatment protocol was determined and scheduled for a three-week period. The treatment protocol was developed by the clinical treatment team. Therapy consisted of 60 minutes of physical therapy (including range of motion exercise, balance training, and gait training) and 60 minutes of occupational therapy (cognitive ability, proprioception) with sessions conducted five days/week during the inpatient program.

**Table 1:** Clinical data (including demographic patient characteristics, diagnosis and tumor localization, history of treatment, and rehabilitation-relevant symptoms and success) BRAF: v-Raf murine sarcoma viral oncogene homolog B; CCNU: lomustine; CNS: central nervous system; ECOG: Eastern Cooperative Oncology Group; F: female; Gy: Gray; IDH: isocitrate dehydrogenase; M: male; MGMT: O6-methylguanine-DNA methyltransferase; no.: number; neg.: negative; PCV: procarbazine hydrochloride, CCNU, and vincristine sulfate; pos.: positive; RCT: radiochemotherapy; STX: stereotactic; TMZ: temozolomide; WHO: World Health Organization

Pat. no.	Gender	Age	Diagnosis	Treatment	Admission status, ECOG	Discharge status, ECOG
1	F	30	Anaplastic astrocytoma CNS WHO 3, IDH pos., MGMT pos., right frontal lobe	Resection, RCT (60 Gy and TMZ)	Fatigue, sleep disturbance, concentration deficits, ECOG 1	Complete recovery, ECOG 0
2	F	47	Anaplastic astrocytoma CNS WHO 3, IDH pos., MGMT pos., left insular	Resection, RCT (60 Gy and TMZ)	Postoperative hemiparesis (4/5) right and dysphasia, ECOG 3	Hemiparesis resolved completely, persistent dysphasia, ECOG 2
3	M	43	Glioblastoma CNS WHO 4, IDH wildtype, MGMT neg., left pontocerebellar	STX biopsy, RCT (60 Gy and TMZ)	Dysbalance, vertigo, nausea, concentration deficits, ECOG 1	Complete recovery, ECOG 0
4	F	44	Glioblastoma CNS WHO 4, IDH wildtype, MGMT neg., right frontal	Resection, RCT (60 Gy and TMZ)	Concentration deficits, ECOG 1	Complete recovery, ECOG 0
5	M	57	Glioblastoma CNS WHO 4, IDH wildtype, MGMT pos., left parietal	Resection, RCT (60 Gy and TMZ and CCNU)	Fatigue, memory deficits, dysthymia, anxiety, ECOG 1	Complete recovery, ECOG 0
6	M	50	Glioblastoma CNS WHO 4, IDH wildtype, MGMT neg., bifrontal	Biopsy, RCT (60 Gy and TMZ)	Fatigue, ECOG 3	Partial recovery, ECOG 2
7	M	51	Glioblastoma CNS WHO 4, IDH wildtype, MGMT neg., right temporal	Resection, RCT (60 Gy and TMZ)	Fatigue, ECOG 2	Complete recovery, ECOG 1
8	F	53	Medulloblastoma CNS WHO 4, cerebellum	Resection, RCT (35 Gy and vincristine)	Ataxia, ECOG 3	Partial recovery, ECOG 2
9	F	53	Oligodendroglioma CNS WHO 2, IDH pos., 1p19d deleted, right parietal	Resection, RCT (54Gy and PCV)	Polyneuropathy, ECOG 2	Partial recovery, ECOG 1
10	M	39	Glioblastoma CNS WHO 4, IDH pos., MGMT pos., right temporal	Resection, RCT (60 Gy and TMZ and CCNU)	Fatigue, ataxia, ECOG 3	Partial recovery, ECOG 2
11	F	62	Glioblastoma CNS WHO 4, IDH wildtype, MGMT neg., left frontal	Resection, RCT (60 Gy and TMZ)	Fatigue, concentration deficits, ataxia, ECOG 3	Partial recovery, ECOG 2
12	M	49	Pleomorphic xanthoastrocytoma CNS WHO 3, MGMT pos., BRAF pos., right temporal	Resection, RCT (60 Gy and TMZ)	Fatigue, ECOG 1	Complete recovery, ECOG 0
13	F	36	Anaplastic oligodendroglioma CNS WHO 3, 1p19q deleted, IDH 1 neg., IDH2 pos., right frontal	Resection, RCT (60 Gy and PCV)	Fatigue, dysthymia, ECOG 1	Complete recovery, ECOG 0
14	M	52	Glioblastoma CNS WHO 4, IDH wildtype, MGMT neg., left temporal	Resection, RCT (60 Gy and TMZ)	Memory deficits, ECOG 2	Partial recovery, ECOG 1
15	M	55	Oligodendroglioma CNS WHO 2, IDH 1 pos., 1p19q deleted, left parietal	Resection, RCT (54 Gy and PCV)	Residual right-sided hemiparesis (4/5), ECOG 2	Complete recovery, ECOG 0
16	M	55	Diffuse pontine glioma CNS WHO 4, IDH wildtype, MGMT neg.	Biopsy, RCT (60 Gy and TMZ)	Memory loss, fatigue ECOG 3	Partial recovery, ECOG 2
17	M	45	Anaplastic astrocytoma CNS WHO 3, IDH pos., MGMT pos., right frontal	Resection, RCT (60 Gy and TMZ)	Concentration deficits, ECOG 1	Complete recovery, ECOG 0
18	M	42	Glioblastoma CNS WHO 4, IDH wildtype, MGMT pos., right frontal	Resection, RCT (60 Gy and TMZ and CCNU)	Concentration deficits, residual left-sided arm weakness (4/5), ECOG 2	Partial recovery, motor strength (5/5), ECOG 1
19	F	59	Glioblastoma CNS WHO 4, IDH wildtype, MGMT pos., right frontal	Resection, RCT (60 Gy and TMZ)	Fatigue, ECOG 2	Complete recovery, ECOG 0
20	F	35	Oligodendroglioma CNS WHO 2, IDH pos., 1p19q deleted, right frontal	Resection	Fatigue, ECOG 1	Complete recovery, ECOG 0
21	M	41	Anaplastic oligodendroglioma CNS WHO 3, IDH pos., MGMT pos.	Resection, RCT (60 Gy and PCV)	Fatigue, ECOG 1	Complete recovery, ECOG 0
22	F	59	Glioblastoma CNS WHO 4, IDH wildtype, MGMT neg., right frontal	Resection, RCT (60 Gy and TMZ)	Dysthymia, fatigue, ECOG 1	Complete recovery, ECOG 0
23	M	35	Astrocytoma CNS WHO 4, IDH pos., MGMT pos., left parietal	Resection, RCT (60 Gy and TMZ)	Residual right-sided hemiparesis (3/5), ECOG 2	Partial recovery (4/5), ECOG 1
24	F	32	Anaplastic astrocytoma CNS WHO 3, IDH pos., MGMT pos., right frontal	Resection, RCT (60 Gy and TMZ)	Fatigue, ECOG 1	Complete recovery, ECOG 0
25	M	35	Oligodendroglioma CNS WHO 2, IDH pos., 1p19q deleted, left temporomesial	Biopsy, PCV	Concentration deficits, ECOG 1	Complete recovery, ECOG 0

Physical therapy consisted of supine-to-sit transition in order to improve mobility, sit-to-stand transfer for the improvement of range and strength of the extremity muscles, standing and gait training in order to improve ataxia, and medical training therapy to combine all sorts of muscular activity and coordination. Occupational therapy included cognitive and proprioceptive therapy consisting of weekly group training sessions. Each training session used exercises to train attention, perception, concentration, and verbal and memory skills.

Psychooncological assistance was offered to each patient three times per week including distress assessment, analysis of individually focused psychological symptoms, and determination of coping strategies. Neuropsychological assistance was performed by verbal and intellectual skills, working memory and mental flexibility, visual and auditory memory, and the analysis of mood and anxiety.

In patients with neurological deficits, the rehabilitative aim was to improve self-management ability. Each patient received regular psychooncological support during the program (three times/week). Neuropathic side effects after chemotherapy were treated with local electrotherapy, local applications of menthol ointments, and the four-cell bathroom and dependent on the severity of symptoms by the prescription of selective serotonin reuptake inhibitors such as duloxetine. Patients with postoperative residual motor deficits received manual treatment. The outcome analysis was calculated based on patient reports using the Eastern Cooperative Oncology Group (ECOG) score at the onset and end of rehabilitation [11]. A two-tailed Student's t-test was applied in order to compare the baseline and discharge ECOG scores to analyze the rehabilitation outcomes [12].

## Results

Twenty-five patients were included in this analysis. Eleven were females (44%), and 14 were males (56%). The median age on admission was 47 years. The interquartile range of the data set was 16.5 years. All patients had primary glial tumors. In the patients with supratentorial localized gliomas, a lateralization tendency towards the right hemisphere was observed. All of these patients featured different neurosurgical histories and neuropathological, especially molecular, diagnoses (Table 1). Four patients had WHO CNS grade 2 tumors (16%), seven had grade 3 tumors (28%), and 14 patients had grade 4 tumors (52%). Seven tumors (31.8%) were localized in the left hemisphere, 15 tumors (68.2%) were localized in the right hemisphere, one tumor (4%) was localized in the cerebellum, and two tumors (8%) were localized in the brainstem. The ECOG scores were determined and compared at the time of admission and discharge: ECOG 0 (n=0 (0%), n=14 (56%)), ECOG 1 (n=12 (48%), n=5 (20%)), ECOG 2 (n=7 (28%), n=6 (24%)), ECOG 3 (n=6 (24%), n=0 (0%)), and ECOG 4 (n=0 (0%), n=0 (0%)).

A two-tailed t-test was applied, where the ECOG scores (on time of admission and on discharge) were compared to each other. The ECOG score on admission was 1.85±0.83 (mean±standard deviation) and on discharge 0.76±0.86. The two-tailed p-value was less than 0.0001. The p-value showed a statistical significance of p<0.05. The physical and psychological impairments were significantly improved at the end of the rehabilitation protocol.

**Figure 2:**
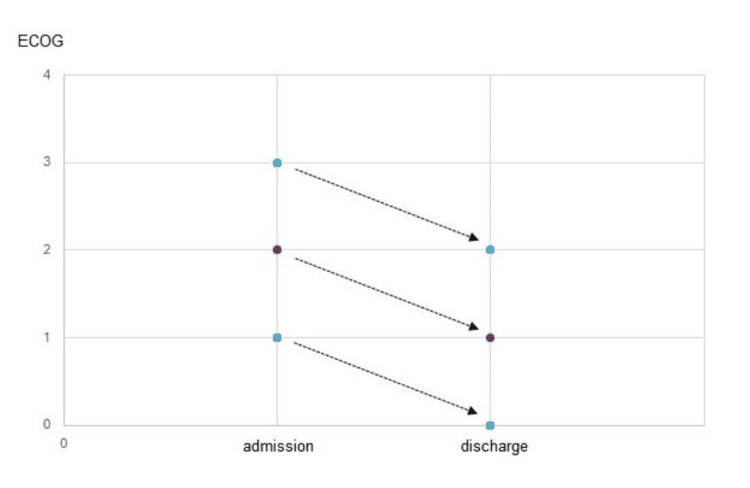
Comparison of ECOG scores between admission and discharge in the rehabilitation period ECOG: Eastern Cooperative Oncology Group

The ECOG scores were compared in each patient at the beginning and the end of rehabilitation. A clear positive outcome of the ECOG scores could be delineated during the rehabilitation process (Figure 2).

## Discussion

The most common primary brain tumors are gliomas [2-4]. Histologically, gliomas can resemble astrocytes, oligodendrocytes, or ependymal cells; thus, on the basis of their morphologic appearance, they are classified as astrocytomas, oligodendrogliomas, or ependymomas, respectively [1]. Astrocytomas express glial fibrillary acidic protein, an intermediate filament found in astrocytes that is routinely used as an aid in classifying a glioma as an astrocytoma [1]. Because astrocytomas and oligodendrogliomas account for the vast majority of gliomas, we focused on these types in this study. The classification of gliomas was updated by the WHO in the year 2021 [1]. Herein, the value of molecular graduation and classification was implemented, which fundamentally influences the postoperative radio-/oncological treatment regime.

Gross total resection in glioma surgery has the most important impact on survival rates [2]. Especially patients undergoing surgery for sub-eloquent or eloquent glioma resections may often suffer from postoperative muscle strength weaknesses and/or neuropsychological deficits, which deteriorate the quality of life and functional independence. Nowadays, microsurgical state-of-the-art treatments include technological adjuncts such as neuronavigation, intraoperative ultrasound and fluorescence with 5-ALA, intraoperative electrophysiological neuromonitoring, and in some centers intraoperative MRI [2].

Physiotherapy is usually recommended for patients with persisting motor deficits, while the inpatient rehabilitation pathway for glioma patients has not been analyzed after the completion of postoperative RCT in detail. There are a few studies dealing with the success of rehabilitation of patients with gliomas, while these articles did not show a statistically significant improvement in the rehabilitation outcome [5,6,9]. Krajewski et al. described significant outcome measures after rehabilitation, in which they examined benign and malignant brain tumor patients directly after surgery in contrast to our study [7]. Physiotherapy is usually recommended for patients with persisting motor deficits, while the inpatient rehabilitation pathway for glioma patients has not been analyzed after the completion of postoperative RCT in detail as in our study. There are different types of physiotherapy including supine-to-sit transition training, sit-to-stand transfer for the improvement of range and strength of the extremity muscles, standing and gait training in order to improve ataxia, and medical training therapy to combine all sorts of muscular activity and coordination. The improvement of functionality is very essential in the rehabilitation of glioma patients, because a better functionality enhances overall survival [13]. The baseline functionality of patients was similar to the baseline characteristics according to the ECOG values of our patient sample.

Rehabilitation treatments cannot be standardized in a dogmatic fashion. They have to be adjusted and focused individually on each patient-dependent symptom and neurological deficits.

The incidence of glioma in Germany is 5-6/100,000 inhabitants [13]. While rehabilitation is also recommended for glioma patients, not all patients are actively advised by the primary neurooncological centers for rehabilitation. This may probably be related to the poor clinical status of the vast majority of patients. We think that compensation of this deficit may possibly raise the number of patients with gliomas for rehabilitation in order to enable the requirements for the realization of a nationwide prospective multicenter study. It is also noteworthy that none of our recruited patients with O6-methylguanine-DNA methyltransferase (MGMT)-positive glioblastomas were treated with tumor-treating fields, while this treatment method should additionally be offered to each eligible patient [14-17].

There might be a bias of hospitalization of glioma patients, who have restrictive neurological and neuropsychological deficits in contrast to asymptomatic patients who are not enrolled in rehabilitation, so that the success of our program can be characterized by a higher clinical quality in the comprehensive treatment of glioma patients.

We could confirm that difficulties related to muscular weakness, coordination, cognitive deficits, and fatigue symptoms were improved within a three-week inpatient rehabilitation program with consecutive exercise and psychooncological assistance. The improvements also optimized dysthymia and provided a positive influence on daily-based activities. The major goals of postoperative rehabilitation include the prevention of complications and the optimization of functional abilities, fast recovery, and fast mobilization, because the improvement of quality of life has a clear effect on the improvement of overall survival. This also enables patients to reintegrate into their former occupation or to reach the capacity for work by occupational redeployment measures.

Limitations

The limitations of our study are based on its retrospective design and limited number of patients, although our clinic is a nationally well-known oncological rehabilitation hospital in the center of Germany. Based on the small sample size of our study, larger prospective studies will be needed to support our statistically significant results.

## Conclusions

Comprehensive inpatient rehabilitation programs enhance the quality of life and may support the integration into the previous occupation of patients after surgery and RCT of high-grade gliomas. By consecutive treatments, motor deficits recover, and mobilization and patient independence can be achieved. This also has a positive impact on existing neuropsychological deficits. We could show that despite the grade of the underlying histological diagnosis, neurooncological rehabilitation can significantly improve quality of life and may contribute to overall survival. Based on the limited number of our patient collective, multicenter studies with broader patient sizes should be performed to confirm our significant results.
